# Incidence of postoperative atrial fibrillation in patients undergoing on-pump and off-pump coronary artery bypass grafting

**DOI:** 10.5935/1678-9741.20150040

**Published:** 2015

**Authors:** Milton Sérgio Bohatch Júnior, Paula Dayana Matkovski, Frederico José Di Giovanni, Romero Fenili, Everton Luz Varella, Anderson Dietrich

**Affiliations:** 1 Universidade Regional de Blumenau (FURB), Blumenau, SC, Brazil.; 2 Hospital Santa Isabel (HSI), Blumenau, SC, Brazil.

**Keywords:** Atrial Fibrillation, Myocardial Revascularization, Postoperative Complications

## Abstract

**Objective:**

To determine the incidence of postoperative atrial fibrillation in patients undergoing on-pump and off-pump coronary artery bypass grafting.

**Methods:**

A retrospective study with analysis of 230 medical records between January 2011 and October 2013 was conducted.

**Results:**

Fifty-six (24.3%) out of the 230 patients were female. The average age of patients undergoing on-pump coronary artery bypass grafting was 59.91±8.62 years old, and off-pump was 57.16±9.01 years old (*P*=0.0213). The average EuroSCORE for the on-pump group was 3.37%±3.08% and for the off-pump group was 3.13%±3% (*P*=0.5468). Eighteen (13.43%) patients who underwent off-pump coronary artery bypass grafting developed postoperative atrial fibrillation, whereas for the onpump group, 19 (19.79%) developed this arrhythmia, with no significant difference between the groups (*P*=0.1955).

**Conclusion:**

Off-pump coronary artery bypass grafting did not reduce the incidence of atrial fibrillation in the postoperative period. Important predictors of risk for the development of this arrhythmia were identified as: patients older than 70 years old and presence of atrial fibrillation in perioperative period in both groups, and non-use of beta-blockers drugs postoperatively in the on-pump group.

## Abstract (pt)

**Objetivo:**

Determinar a incidência de fibrilação atrial no pós -operatório de pacientes submetidos à cirurgia de revascularização do miocárdio com e sem circulação extracorpórea.

**Métodos:**

Foi realizado um estudo retrospectivo com análise de 230 prontuários entre janeiro de 2011 e outubro de 2013.

**Resultados:**

Do total de 230 pacientes, 56 (24,3%) eram do sexo feminino. A média de idade dos pacientes submetidos à cirurgia de revascularização do miocárdio com circulação extracorpórea foi de 59,91±8,62 anos, e sem circulação extracorpórea, foi de 57,16±9,01 anos (P=0,0213). A média do Euroscore para o grupo com circulação extracorpórea foi de 3,37%±3,08% e para o grupo sem circulação extracorpórea foi de 3,13%±3% (P=0,5468). Entre os pacientes que realizaram a cirurgia de revascularização do miocárdio sem circulação extracorpórea, 18 (13,43%) desenvolveram fibrilação atrial no pós-operatório, enquanto no grupo com circulação extracorpórea, 19 (19,79%) desenvolveram esta arritmia, não havendo diferença significativa entre os grupos (P=0,1955).

**Conclusão:**

A cirurgia de revascularização do miocárdio sem circulação extracorpórea não diminuiu a incidência de fibrilação atrial no período pós-operatório. Identificamos como preditores de risco para o desenvolvimento desta arritmia: idade superior a 70 anos e presença de fibrilação atrial no período transoperatório em ambos os grupos estudados, e o não uso de medicamentos betabloqueadores no pós-operatório do grupo com circulação extracorpórea.

**Table:** 

**Abbreviations, acronyms & symbols**	
AF	Atrial fibrillation
ARF	Acute renal failure
CABG	Coronary artery bypass grafting
CCU	Coronary care unit
COPD	Chronic obstructive pulmonary disease
CPB	Cardiopulmonary bypass
CRF	Chronic renal failure
DM	Diabetes mellitus
ECG	Ele ctroc ardi ograms
FURB	Regional University of Blumenau
ICU	Intensive care units
PTCA	Percutaneous transluminal angioplasty
PVD	Peripheral vascular disease

## INTRODUCTION

Atrial fibrillation (AF) is a common complication after cardiac surgery, affecting about 10% to 40% of patients undergoing Coronary Artery Bypass Grafting (CABG)^[1-3]^. This arrhythmia occurs most frequently in the first five days of the postoperative period, peaking between 24 and 72 hours, being uncommon after the first week^[3,4]^.

The postoperative AF represents a four-fold increased risk of stroke in comparison to the evolution of patients who remain in sinus rhythm^[5]^. It is meaningfully associated with postoperative complications, such as heart failure, hypoxia, hypovolemia, sepsis and electrolyte disturbances^[6]^, as well as it doubles the overall mortality rate in the postoperative period^[7]^. Such complications increase significantly the cost of surgical treatment by extending the hospital stay, present higher readmission in Intensive Care Units (ICU), prolong the duration of mechanical ventilation, increase the need for vasoactive drugs, ventricular assist devices and even reintubation^[8]^.

The onset of AF after cardiac surgery is associated with preoperative, intraoperative and postoperative factors^[9]^. Among the preoperative clinical findings, advanced age is considered an independent predictor of AF^[2,3]^, present in about 50% of patients older than 80 years-old^[4]^. The intraoperative factors can be consequent to cardiac ischemia and inflammation inherent to the complexity of the surgical technique. In these situations, the time of aortic clamping, handling and atrial cannulation and the use of cardiopulmonary bypass (CPB) are reported as factors that support the installation of AF^[8]^. The CABG with CPB (on-pump CABG) appears to be associated with a higher incidence of AF in the postoperative compared to off-pump CABG. However, some studies reported no relevant difference in the incidence of this arrhythmia regarding the use or not of CPB^[4]^. Some of the conditions associated with the onset of AF after surgery include: infections, prolonged mechanical ventilation, hemodynamic instability, myocardial ischemia and low cardiac output in the postoperative period^[10]^.

The prevalence of AF, its impact on the health system costs and the patients' recovery justify the demand for clarification of triggers and therapeutic procedures to reduce AF in the postoperative period. In this context, the off-pump CABG has been explored as an emerging technique that can reduce the adverse effects of the on-pump technique, which predisposes to AF. The number of off-pump procedures performed has increased significantly in recent years. However, definitive conclusions about the incidence of AF in on-pump and in off-pump CABG surgeries have not been well established yet^[11,12]^.

The objective of this study is to determine the incidence of AF in the postoperative period of patients undergoing on-pump and off-pump CABG and to identify predictors of risk associated with the development of this arrhythmia.

## METHODS

A retrospective cohort study was conducted with 230 consecutive medical records of patients that underwent on-pump and off-pump CABG from January 2011 to October 2013 in the Heart Surgery Service of Santa Isabel Hospital, in Blumenau, Santa Catarina, Brazil. The respective protocol was approved by the Ethics Committee on Human Research of the Regional University of Blumenau (FURB) under number 203/12.

Inclusion criteria:

- All patients undergoing elective CABG.
Exclusion criteria:
· Chronic AF and/or other cardiac arrhythmias documented.
· Heart valve disease.
· Emergency surgery.
· Cardiac reoperation.

They were divided in two groups: patients undergoing on-pump CABG (96 patients) and patients undergoing off -pump CABG (134 patients). Data were grouped in preoperative, intraoperative, and postoperative variables. Subsequently, the variables were evaluated in a comparative way.

The preoperative variables were: age, sex, smoking, Diabetes Mellitus (DM), hypertension, dyslipidemia, Peripheral Vascular Disease (PVD), use of medications (statins and/or beta-blockers), Chronic Obstructive Pulmonary Disease (COPD), extra cardiac artery disease, Acute Renal Failure (ARF) or Chronic Renal Failure (CRF), neurological dysfunction, prior and acute Percutaneous Transluminal Angioplasty (PTCA) or chronic coronary insufficiency. The EuroSCORE was calculated for all patients.

The perioperative variables were: complications (increased bleeding, hemodynamic instability, arrhythmias, need for pacemaker and/or intra-aortic balloon, acute myocardial infarction), number of grafts, the need for transfusion (packed red blood cells, fresh frozen plasma and platelets), use of vasoactive drugs (noradrenaline, dobutamine, dopamine, nitroglycerin and/or sodium nitroprusside), CPB time (in minutes), aortic clamping time (in minutes) and mortality. It is important to emphasize that each case was individually analyzed for the need of blood transfusion, taking into account the hemodynamic conditions, the volume of bleeding, the patient's age and history of bleeding disorders. Generically, the bleeding was considered significant when the delivery of chest tubes in the immediate postoperative period was 150 mL/h for more than 3 consecutive hours, or output rate greater than 500 mL in 6 hours, or output rate greater than 1000 mL in 24 hours.

The postoperative variables were: Coronary Care Unit (CCU) time, hospital stay, use of vasoactive drugs (noradrenaline, dobutamine, dopamine, nitroglycerin and/or sodium nitroprusside), use of medications (statins and/or beta-blockers) and need for blood products. The complications in the CCU considered were: increased bleeding, need for reoperation, infection (in the surgical wound, pneumonia and/or sepsis), neuropsychiatric (ischemic stroke, delirium and/or altered levels of consciousness), ARF, acute abdomen, pneumothorax, haematological disorders (anemia and/or thrombocytopenia), cardiac disorders (hemodynamic instability, pericarditis, cardiorespiratory arrest, myocardial infarction, circulatory shock and/or use of intra-aortic balloon) and mortality.

The analyzed outcome was the incidence of postoperative AF in on-pump and off-pump CABG. The presence of AF in the postoperative period was determined by analysis of electrocardiograms (ECG) present in the medical records. All patients undergoing CABG in this study underwent 12-lead ECG in the immediate postoperative period, every 24 hours during hospitalization in CCU and in the infirmary, and 24 hours prior to discharge.

Data were organized in descriptive tables containing: frequency, mean and standard deviation. The average and proportions estimate were made with a 95% confidence interval. In order to compare the frequencies within the same distribution, it was used the Chi-Squared test of adherence. For the association of categorical variables with the groups, it was used Chi-Squared test of independence in cases of frequencies equal to or greater than 5, and Fisher's exact test, in cases of frequencies below 5. For the analysis of continuous variables the Student t test was used. In all tests, the significance is considered when *P* value < 0.05. Data analysis was performed on Microsoft Excel 2010 software and EpiInfo version 7, 2012.

The off-pump CABG has become the preferred technique at this service since 2008. It is currently used in approximately 80% of CABG surgery. During the study period, the main criterion used to display on-pump and off-pump CABG was the experience of surgeons. Regarding the surgical data, all patients were submitted to balanced general anesthesia. In the off-pump group, 362 arterial and venous grafts were performed, and about 3 grafts per patient. In this technique, no drug was used for myocardial protection and reperfusion with "bypass" was held only in the proximal right coronary anastomoses. In the on-pump group, 275 arterial and venous grafts were performed, and about 3 grafts per patient. Nipro oxygenator and Saint Thomas Braille cardioplegia for myocardial protection were used. The patients in this group underwent systemic hypothermia 30-32ºC.

The preoperative variables are shown in Table 1. Fifty-six (24.3%) out of the 230 patients, were female and 174 (75.7%) were male. The average age of patients undergoing on-pump CABG was 59.91±8.62 years-old and off-pump was 57.16±9.01 years-old (*P*=0.0213). Thirty-five patients were younger than 50 years-old, 26 (19.4%) from the group that underwent off-pump surgery and 9 (9.4%) from the group undergoing on-pump CABG, with significant differences between both groups (*P*=0.0360). The other preoperative variables are equally distributed between the groups (*P*>0.05). The average EuroSCORE for the on-pump group was 3.37%±3.08% and for the off-pump group was 3.13%±3% (*P*=0.5468).

**Table 1:** Preoperative variables of patients undergoing on-pump and off-pump coronary artery bypass grafting.

Technical Features	On-Pump (n=96)	Off-Pump (n=134)	*P* Value
Age (years) (mean±SD)	(59.91±8.62)	(57.16±9.01)	0.0213
Age < 50	9 (9.4%)	26 (19.4%)	0.0360
50 ≤ i < 60	43 (44.8%)	52 (38.8%)	0.3632
60 ≤ i < 70	31 (32.3%)	45 (33.6%)	0.8374
70 ≤ i < 80	11 (11.5%)	10 (7.5%)	0.2995
Age ≥ 80	2 (2.1%)	1 (0.7%)	-
Female patients	23 (24.2%)	33 (24.6%)	0.9424
EuroSCORE	(3.37%±3.08%)	(3.13%±3%)	0.5468
Statins	87 (90.6%)	120 (89.6%)	0.7891
Beta-blocker	87 (90.6%)	111 (82.8%)	0.0923
Chronic coronary insufficiency	45 (46.9%)	73 (54.5%)	0.2553
Prior percutaneous angioplasty	30 (31.25%)	36 (26.87%)	0.4685
Diabetes Mellitus	30 (31.25%)	47 (35.07%)	0.5444
Hypertension	73 (76.04%)	107 (79.85%)	0.4898
Dyslipidemia	64 (66.67%)	86 (64.18%)	0.6961
Peripheral vascular disease	4 (4.17%)	9 (6.72%)	0.4089
Cerebrovascular disease	6 (6.25%)	11 (8.21%)	0.5755
Arteriopathy extracardiac	61 (63.54%)	73 (54.48%)	0.1692
Acute or chronic renal failure	6 (6.25%)	8 (5.97%)	0.9302
Chronic obstructive pulmonary disease	8 (8.3%)	13 (9.7%)	0.7224
Smokers	23 (24%)	30 (22.4%)	0.2539

## RESULTS

The intraoperative variables are shown in Table 2. It was observed a higher consumption of blood products in the on-pump when compared to off-pump CABG (64.58% vs. 20.9%; *P*<0.001), mainly represented by red blood cell units (2.35±0.89 in the on-pump group and 1.71±0.73 in the off-pump group; *P*=0.0164). Off-pump group showed higher tendency to develop AF, however there was no statistical difference between groups (*P*=0.0560). The other intraoperative variables are equally distributed between the groups (*P*>0.05) and there were no deaths in this period.

**Table 2:** Intraoperative variables of patients undergoing on-pump and off-pump coronary artery bypass grafting.

Technical Features	On-Pump (n=96)	Off-Pump (n=134)	*P* Value
Number of grafts (Mean±SD)	(2.86±0.75)	(2.7±0.86)	0.1357
Blood products	62 (64.58%)	28 (20.9%)	0.0000
Packed red blood cells (units)	(2.35±0.89)	(1.71±0.73)	0.0164
Fresh frozen plasma (units)	(2.74±0.89)	(2.42±0.51)	0.0957
Platelets (units)	(8.6±3.66)	(7.33±2.52)	0.5911
Sympathomimetic drugs	41 (42.71%)	58 (43.28%)	0.9308
Dobutamine	26 (27.08%)	40 (30.08%)	0.6218
Noradrenaline	11 (11.46%)	17 (12.78%)	0.7629
Dopamine	10 (10.42%)	10 (7.46%)	0.4330
Vasodilators drugs	22 (22.92%)	29 (21.64%)	0.8185
Nitroglycerine	7 (7.29%)	12 (8.96%)	0.6513
Sodium nitroprusside	18 (18.75%)	18 (13.53%)	0.2846
Complications	27 (28.13%)	46 (34.33%)	0.3189
Increased bleeding	5 (5.21%)	6 (4.48%)	0.7979
Hemodynamic instability	13 (13.54%)	20 (14.93%)	0.7678
Arrhythmias	9 (9.38%)	23 (17.16%)	0.0923
Atrial fibrillation	1 (1.04%)	8 (6.02%)	0.0560
Pacemaker	2 (2.08%)	1 (0.75%)	0.3781
Intra-aortic balloon	-	3 (2.24%)	0.1400
Acute myocardial infarction	-	3 (2.24%)	0.1400
Mortality	-	-	-

Postoperative variables are shown in Table 3. The group undergoing on-pump CABG showed a higher incidence of anemia, when compared to the off-pump group (27.08% vs. 16.42%, *P*=0.0497). On-pump group also presented major bleeding when compared to off-pump group (12.5% vs. 2.99%, *P*=0.0052). For other events there was no statistical difference (*P*>0.05).

**Table 3:** Postoperative variables of patients undergoing on-pump and off-pump coronary artery bypass grafting.

Technical Features	On-Pump (n=96)	Off-Pump (n=134)	*P* Value
Atrial Fibrillation	19 (19.79%)	18 (13.43%)	0.1955
Sympathomimetic drugs	57 (59.38%)	76 (56.72%)	0.6872
Dobutamine	28 (29.17%)	43 (32.09%)	0.6361
Noradrenaline	43 (44.79%)	48 (35.82%)	0.1701
Dopamine	3 (3.13%)	4 (2.99%)	0.9514
Adrenalin	1 (1.04%)	-	0.2364
Vasodilators drugs	43 (44.79%)	63 (47.01%)	0.7387
Nitroglycerine	28 (29.17%)	53 (39.55%)	0.1039
Sodium nitroprusside	25 (26.04%)	25 (18.66%)	0.1806
Statins	91 (94.79%)	127 (94.78%)	0.9958
Beta-blocker	85 (88.54%)	118 (88.06%)	0.9108
Blood products	27 (28.13%)	25 (18.66%)	0.0905
Complications coronary care unit			
Increased bleeding	12 (12.5%)	4 (2.99%)	0.0052
Reoperation	-	1 (0.75%)	0.3963
Infectious complications	8 (8.33%)	8 (5.97%)	0.4873
Wound infection (chest)	2 (2.1%)	1 (0.7%)	0.3781
Bronchopneumonia	5 (5.21%)	7 (5.22%)	0.9958
Sepsis	2 (2.08%)	1 (0.75%)	0.3781
Delirium/altered levels of consciousness	7 (7.29%)	12 (8.96%)	0.6513
Ischemic stroke	1 (1.04%)	2 (1.49%)	0.7663
Acute renal failure	4 (4.17%)	2 (1.49%)	0.2096
Acute abdomen	2 (2.08%)	-	0.0933
Pneumothorax/Pneumomediastinum	2 (2.08%)	4 (2.99%)	0.6722
Anemia	26 (27.08%)	22 (16.42%)	0.0497
Thrombocytopenia	1 (1.04%)	3 (2.24%)	0.4934
Hemodynamic instability	10 (10.42%)	7 (5.22%)	0.1377
Pericarditis	1 (1.04%)	4 (2.99%)	0.3189
Cardiopulmonary arrest	4 (4.2%)	4 (3%)	0.6296
Acute myocardial infarction	1 (1.04%)	2 (1.49%)	0.7663
Circulatory shock	4 (4.17%)	3 (2.24%)	0.4013
Intra-aortic balloon	-	1 (0.75%)	0.3963
Time of Coronary Care Unit (days)	(3.21±2.23)	(3.22±2.7)	0.9619
Length of hospital stay (days)	(8.19±7.35)	(7.93±3.99)	0.7513
Mortality	6 (6.3%)	3 (2.2%)	0.1218

In the off-pump group, 18 (13.43%) patients developed AF in the postoperative period, while in the on-pump group, 19 (19.79%) developed this arrhythmia, with no significant difference (*P*=0.1955).

Relations between the main risk factors for the development of AF in the postoperative period and the occurrence of this arrhythmia in the groups studied are described in Tables 4 and 5.

**Table 4:** Association of the variables with groups that have and have not developed postoperative atrial fibrillation in patients undergoing off-pump CABG.

Variables	Developed atrial fibrillation (n=18)	Not developed atrial fibrillation (n=116)	*P* Value
Preoperative			
Female	6 (33.3%)	27 (23.3%)	0.3568
Age (> 70 years)	4 (22.2%)	5 (4.3%)	0.0047
Hypertension	5 (27.8%)	22 (19%)	0.3858
Diabetes Mellitus	9 (50%)	38 (32.8%)	0.1538
Chronic obstructive pulmonary disease	1 (5.6%)	12 (10.3%)	0.5230
Non-use of beta-blocker	6 (33.3%)	17 (14.7%)	0.0505
Non-use of statins	2 (11.1%)	12 (10.3%)	0.9212
Intraoperative			
Blood products	5 (27.8%)	23 (19.8%)	0.4402
Arrhythmias	4 (22.2%)	19 (16.4%)	0.5408
Atrial fibrillation	3 (16.7%)	5 (4.3%)	0.0410
Ventricular fibrillation	3 (16.7%)	7 (6.1%)	0.1135
Ventricular extrasystole	0 (0%)	2 (1.7%)	0.5729
Sinus tachycardia	0 (0%)	2 (1.7%)	0.5729
Postoperative			
Time of Coronary Care Unit (days)	(4.72±3.32)	(2.91±2.25)	0.0374
Length of hospital stay (days)	(9.89±4.09)	(7.62±3.91)	0.0244
Non-use of beta-blocker	2 (11.1%)	14 (12.1%)	0.9072
Non-use of statins	2 (11.1%)	5 (4.3%)	0.2276
Vasoactive drugs	13 (72.2%)	63 (54.3%)	0.1536
Infectious complications	2 (11.1%)	6 (5.2%)	0.3225
Mortality	0 (0%)	3 (2.6%)	0.4902

**Table 5:** Association of the variables with groups that have and have not developed postoperative atrial fibrillation in patients undergoing on-pump CABG.

Variables	Developed atrial fibrillation (n=19)	Not developed atrial fibrillation (n=77)	*P* Value
Preoperative			
Female	5 (26.3%)	18 (23.7%)	0.8107
Age (> 70 years)	6 (31.6%)	5 (6.5%)	0.0021
Hypertension	8 (42.1%)	16 (20.8%)	0.0545
Diabetes Mellitus	5 (26.3%)	25 (32.5%)	0.6044
Chronic obstructive pulmonary disease	2 (10.5%)	6 (7.8%)	0.6994
Non-use of beta-blocker	0 (0%)	9 (11.7%)	0.1175
Non-use of statins	1 (5.3%)	8 (10.4%)	0.4923
Intraoperative			
Aortic clamping time	(67.11±17.18)	(69.41±21.26)	0.66299
Cardiopulmonary bypass time	(100±23.69)	(104.41±26.6)	0.51121
Blood products	12 (63.2%)	50 (64.9%)	0.8847
Arrhythmias	2 (10,5%)	7 (9.1%)	0.8476
Atrial fibrillation	1 (5.3%)	0 (0%)	0.0430
Ventricular fibrillation	1 (5.3%)	3 (3.9%)	0.7894
Ventricular extrasystole	0 (0%)	1 (1.3%)	0.6175
Sinus tachycardia	0 (0%)	0 (0%)	-
Postoperative			
Time of Coronary Care Unit (days)	(4.53±3.11)	(2.89±1.69)	0.0652
Length of hospital stay (days)	(10.26±11.26)	(7.68±6.01)	0.3440
Increased bleeding	2 (10.5%)	3 (3.9%)	0.2441
Non-use of beta-blocker	5 (26.3%)	6 (7.8%)	0.0232
Non-use of statins	2 (10.5%)	3 (3.9%)	0.2441
Vasoactive drugs	14 (73.7%)	43 (55.8%)	0.1562
Infectious complications	4 (21.1%)	4 (5.2%)	0.0251
Mortality	4 (21.1%)	2 (2.6%)	0.0029

Among the preoperative risk factors, age over 70 years old is strongly associated with the development of AF in both groups (22.2%; *P*=0.0047 in the off-pump group and 31.6% in the on-pump group; *P*=0.0021). There was a tendency in developing this arrhythmia in the on-pump group patients with hypertesion (42.1%; *P*=0.0545), although this was not statistically significant. Similarly, analyzing the non-use of beta-blocker drugs, the off-pump group had a tendency to develop AF (33.3%; *P*=0.0505).

Amongst the intraoperative risk factors, only the presence of AF during surgery showed relation to the development of this arrhythmia in the postoperative period in both groups, on-pump CABG (5.3%; *P*=0.0430) and off-pump (16.7%; *P*=0.0410).

Amongst the postoperative risk factors in the off-pump group, CCU time and hospital stay were remarkably higher in patients who developed AF (4.72±3.32; *P*=0.0374 and 9.89±4.09; *P*=0.0244, respectively) when compared to those who did not develop this arrhythmia. Analyzing the non-use of beta-blocker drugs, there was a strong association with the development of AF in the on-pump group (26.3%; *P*=0.0232). This group showed expressive relationship between the presence of infectious complications and the development of this arrhythmia (21.1%; *P*=0.0251).

The overall mortality of 230 patients undergoing CABG was 3.91%. Six (6.3%) out of the 96 patients in the on-pump group died, compared to 3 (2.2%) out of 134 patients in the off-pump group (P=0.1218). Mortality in the on-pump group was notably higher in patients who developed AF when compared to patients who did not develop the arrhythmia (21.1%; *P*=0.0029). No deaths were observed in patients undergoing off-pump CABG that developed postoperative AF.

## DISCUSSION

Atrial fibrillation is the most common arrhythmia in the postoperative period of patients undergoing CABG^[13,14]^. The presence of this arrhythmia is associated with longer clinical recovery time, besides being able to worsen the hemodynamic state of the patient, to increase the risk of congestive heart failure and trigger thromboembolic events^[5,14,15]^. The increased morbidity and the consequent prolongation of hospitalization significantly increase the hospital costs^[14,15]^.

Unlike non-surgical cases, the AF in the postoperative period does not have a well-defined cause, but recent studies suggest multifactorial mechanism, involving: oxidative stress, inflammation, atrial fibrosis, changes in autonomic tonus and connexin expression, that increase the dispersion of atrial refractoriness and favor the formation of a proarrhythmic substrate^[16]^.

Some risk factors are related to increased incidence of AF in the postoperative period of cardiac surgery. Among these factors are: paroxysmal AF history, previous myocardial infarction, DM, hypertension, COPD, discontinuation of beta-adrenergic drugs preceding surgery, aortic clamping time, postoperative ischemia and vasoactive amines^[16]^. Advanced age is one of the most important factors being considered an independent predictor of this arrhythmia after CABG^[2,16]^.

Studies showed the use of CPB as an important risk factor involved in the development of AF, which can be explained by the resulting ischemia of cardioplegia, the CPB time, as well as the cannulation and aortic clamping^[4,13,17]^. In addition, blood contact with non-physiological surfaces of CPB machine results in an inflammatory response that may also be involved in the development of AF^[4,13,17]^. In view of these aspects, it is expected that off-pump CABG be associated with a lower incidence of AF in the postoperative period. Although some studies show such benefit^[13]^, they are still controversial.

In a comparative study between on-pump and off-pump CABG, the on-pump group had major complications, such as stroke, reoperation for bleeding, prolonged mechanical ventilation, AF and period of hospitalization^[18]^. Some well-designed clinical trials comparing the two techniques showed no significant difference in the incidence of arrhythmias, especially of AF^[15,19]^.

In this study, the overall incidence of AF after CABG was 16.1%, similar that found in other studies^[14,16]^. However, there was no significant difference in the incidence of AF between groups, being 13.43% in the off-pump group and 19.79% in the on-pump group (*P*=0.1955). These results were similar those found in other studies^[14,15]^.

Lin et al.^[14]^ observed that off-pump CABG also did not reduce the incidence of postoperative AF. The authors believe that the results can be explained by the greater use of beta-blockers in the preoperative period for the on-pump group. It is believed that the use of beta-blockers preoperatively is an effective measure for the prophylaxis of AF^[2,12,16]^. In our study, the on-pump group also showed greater use of beta-blocker drugs preoperatively, compared to the off-pump group (90.6% and 82.8%, respectively), but these results were not statistically significant (*P*=0.0923).

A notable increase in cases of AF in patients over 70 was identified, regardless of the method used for myocardial revascularization, and it is the preoperative variable statistically relevant among the analyzed. It is estimated that for each decade of life, there is a 75% increase in the odds of developing AF in the postoperative period^[20]^. Thus, anyone over 70 has a high risk of developing AF^[20]^. Aging causes degenerative changes in the atrial myocardium, leading to changes in the electrical characteristics of the sinus and AV nodes, which contributes to fragment the impulse propagation^[21]^. In these patients, the atrium is generally dilated, hypertrophic and/or with fibrosis areas, compromising the structure and function of the sinus node^[16]^. Often, patients older than 70 years-old are carriers of atherosclerosis, hypertension and/or diabetes mellitus, in addition to having reduced cardiopulmonary reserve as to the younger, contributing greatly to the genesis of AF^[16]^.

In perioperative period, we found in both groups, the occurrence of AF in this period is a predictor for recurrence postoperatively, which is not observed in other analyzed arrhythmias. It is known that prior AF is a risk factor for development of AF in the postoperative period^[4,16]^. It is speculated that vulnerable individuals with AF, present electrophysiological pro-arrhythmic substrate, which is then exacerbated by surgery^[4]^. Therefore, the occurrence of AF during surgery could expose susceptible individuals with great potential for recurrence postoperatively. Also, the arrhythmia during surgery can result from individuals with paroxysms of AF with spontaneous resolution which were undiagnosed.

In the postoperative period, we observed the association of non-use of beta-blocker drugs and the development of postoperative AF in on-pump group. Some studies^[3,21,22]^ showed beta-blockers are effective in preventing postoperative AF. It is believed that the increased sympathetic tonus may predispose patients to the development of AF, and therefore, beta-blockers can prevent this arrhythmia to modulate this pathway^[13]^.

Previous studies have shown that the presence of AF postoperative can increase hospitalization in 2-4 days, thus increasing the cost of treatment^[23]^. This study showed that the development of postoperative AF in the off-pump CABG is associated with increased length of stay in the CCU and longer total hospitalization compared to patients who did not develop AF. However, there was no statistical difference in mortality to the same group. The on-pump group showed no significant difference in the length of stay, but showed higher mortality in patients who developed AF compared to those who did not develop the arrhythmia. This can be explained by the fact that patients undergoing CABG with CPB had higher rates of infectious complications.

The overall mortality in this study was 3.91%, comparable to the study of Sá et al.^[24]^ which showed a mortality of 3.5% for patients classified as moderate risk (EuroSCORE 3-5). As well as the results of the study of Shroyer et al.^[25]^, we found no statistical difference in mortality between on-pump and off-pump CABG.

## CONCLUSION

Off-pump CABG did not reduce the incidence of AF in the postoperative period. We identified as predictors of risk for developing this arrhythmia: patients older than 70 years-old and the presence of AF in the perioperative period in both groups, and non-use of beta-blocker drugs postoperatively in on-pump group.

**Table:** 

**Authors’ roles & responsibilities**	
MSBJ	Analysis and/or interpretation of data; implementation of projects and/or experiments; manuscript writing or critical review of its content
PDM	Analysis and/or interpretation of data; implementation of projects and/or experiments; manuscript writing or critical review of its content
FJDG	Final approval of the manuscript; manuscript writing or critical review of its content
RF	Study design
ELV	Final approval of the manuscript
AD	Final approval of the manuscript; study design; manuscript writing or critical review of its content
